# Integrated Physiological and Transcriptomic Analyses of *Saccharomyces cerevisiae* Under Syringaldehyde Stress

**DOI:** 10.3390/biology15161410

**Published:** 2026-08-17

**Authors:** Xiufeng Long, Xinru Li, Xuemei Zhao, Yupeng Du, Fuxing Niu, Yi Yi

**Affiliations:** 1 College of Biological and Chemical Engineering, Guangxi University of Science and Technology, Liuzhou 545006, China; longxiuf@163.com (X.L.);; 2 Guangxi Key Laboratory of Green Processing of Sugar Resources, Guangxi University of Science and Technology, Liuzhou 545006, China; 3 State Key Laboratory of Non-Food Biomass Energy Technology, Nanning 530000, China

**Keywords:** *Saccharomyces cerevisiae*, syringaldehyde, ethanol fermentation, cell-envelope integrity, transcriptomics, pentose phosphate pathway

## Abstract

Syringaldehyde is a major lignin-derived inhibitor in lignocellulosic hydrolysates that affects growth, metabolism, and ethanol fermentation in *Saccharomyces cerevisiae*; however, the mechanisms underlying its toxicity and cellular adaptation remain poorly understood. In this study, fermentation analysis, physiological characterization, and transcriptomic profiling were integrated to investigate the response of *S. cerevisiae* to syringaldehyde stress. Syringaldehyde inhibited yeast growth and ethanol fermentation in a concentration-dependent manner. At 1.4 g/L, it caused only a minor decrease in final optical density at 560 nm (OD_560_) (2.35%) but markedly decreased ethanol production and total sugar fermentation efficiency (18.65% and 17.64%, respectively), accompanied by delayed early-stage sugar utilization. Physiological analyses demonstrated that syringaldehyde induced cell-envelope alterations and induced membrane lipid peroxidation, whereas intracellular glycerol accumulation occurred only after prolonged exposure, indicating a delayed adaptive response. Transcriptome analysis identified 496 differentially expressed genes, with repression of ribosome biogenesis and cofactor biosynthesis and activation of the pentose phosphate pathway and aromatic aldehyde detoxification. Notably, *ADH7*, *GND2*, and *TKL2* were strongly induced, suggesting enhanced NADPH-dependent detoxification. Collectively, these findings demonstrate that syringaldehyde induces coordinated physiological stress responses and metabolic reprogramming, including alterations in cell-envelope integrity, oxidative imbalance, and changes in fermentation-associated pathways, providing mechanistic insights into yeast adaptation to lignin-derived aromatic aldehyde stress and identifying potential targets for engineering more robust industrial yeast strains.

## Simple Summary

Renewable ethanol production from plant biomass is limited by inhibitory compounds generated during biomass processing. Syringaldehyde is a lignin-derived compound that reduces yeast fermentation efficiency, but the mechanisms underlying its toxicity and yeast adaptation remain unclear. In this study, we investigated how syringaldehyde affects *Saccharomyces cerevisiae* by combining fermentation analysis, cellular characterization, and gene expression analysis. We found that syringaldehyde strongly impaired ethanol production while only slightly affecting biomass accumulation, indicating that it primarily disrupted fermentative function rather than causing extensive growth inhibition. Further analyses showed that syringaldehyde induced multiple physiological stress responses, including alterations in cell envelope and increased membrane lipid oxidation. At the molecular level, yeast cells reduced the expression of genes related to protein production and cellular growth, while activating pathways involved in generating cellular reducing power and removing harmful aldehydes. These responses indicate that yeast cells adapt to syringaldehyde stress by reallocating metabolic resources from growth and ethanol production toward cellular protection and detoxification. This study improves understanding of microbial adaptation to lignin-derived aromatic aldehydes and provides insights for developing more robust yeast strains for sustainable bioethanol production.

## 1. Introduction

The increasing demand for sustainable energy and the depletion of fossil fuel resources have driven extensive interest in the production of bioethanol from renewable lignocellulosic biomass. Owing to its abundance, low cost, and non-competition with food resources, lignocellulosic biomass derived from agricultural residues, forestry wastes, and dedicated energy crops is considered one of the most promising feedstocks for second-generation bioethanol production [1]. However, the inherent recalcitrance of lignocellulosic biomass necessitates physicochemical or biological pretreatment to improve enzymatic hydrolysis and sugar release [2]. Although this process enhances biomass deconstruction and fermentable sugar availability, it inevitably generates a variety of inhibitory compounds, including furan derivatives, weak organic acids, and phenolic compounds, which severely impair microbial growth and ethanol fermentation performance [3]. Therefore, overcoming inhibitor-mediated stress has become a critical challenge for improving the efficiency of lignocellulosic bioconversion.

Among the inhibitors generated during lignocellulosic pretreatment, lignin-derived phenolic compounds are considered particularly detrimental to microbial fermentation. Low-molecular-weight aromatic aldehydes, including vanillin, p-hydroxybenzaldehyde, and syringaldehyde, are generally regarded as among the most toxic phenolic inhibitors present in hydrolysates [4]. Syringaldehyde, a syringyl-derived aromatic aldehyde released from lignin depolymerization, has attracted considerable attention due to its inhibitory effects on microbial growth and fermentation performance. The concentration of syringaldehyde reported in lignocellulosic hydrolysates varies substantially depending on biomass type and pretreatment strategy. For example, syringaldehyde concentrations of approximately 55 mg/L, 107 mg/L, and 213 mg/L have been reported in dilute-acid-pretreated corn stover hydrolysate, spruce hydrolysate, and hemicellulose hydrolysate, respectively, indicating that syringaldehyde is generally present at sub-gram-per-liter levels in practical hydrolysates [5,6,7]. Syringaldehyde has been recognized as a potent lignin-derived inhibitor affecting microbial growth and fermentation performance. For instance, Cortez and Roberto reported that syringaldehyde inhibited cell growth and xylitol production in *Candida guilliermondii*, demonstrating its inhibitory potential as a lignin-derived aromatic aldehyde [8]. Similarly, Kelly et al. showed that syringaldehyde exposure significantly reduced yeast growth and impaired ethanol fermentation performance [9]. However, previous studies have mainly focused on phenotypic inhibition of growth and fermentation, whereas the physiological damage and molecular mechanisms underlying yeast adaptation to syringaldehyde stress remain insufficiently understood.

The toxicity of aromatic aldehydes is associated with multiple interconnected cellular targets and regulatory processes. Previous studies have demonstrated that these compounds can compromise membrane integrity, disrupt intracellular redox homeostasis, induce oxidative stress, impair protein synthesis, and perturb central carbon metabolism in *Saccharomyces cerevisiae* [10,11,12,13]. In addition, the electrophilic aldehyde group can readily react with intracellular nucleophiles, including proteins and nucleic acids, thereby interfering with essential cellular functions and metabolic activities [10].

Increasing evidence suggests that the toxicity of aromatic aldehydes extends beyond direct cellular damage and is closely linked to disturbances in intracellular redox metabolism. In *S. cerevisiae*, aromatic aldehydes are primarily detoxified through NAD(P)H-dependent reduction reactions catalyzed by alcohol dehydrogenases and aldehyde reductases, converting toxic aldehydes into their corresponding less toxic alcohols [14]. While this detoxification process alleviates inhibitor toxicity, it imposes a substantial demand on intracellular reducing equivalents. Because ethanol production, antioxidant defense, and biomass synthesis also depend heavily on NADH and NADPH availability, aldehyde detoxification may compete with normal cellular metabolism for reducing power [15]. Thus, syringaldehyde stress may require yeast cells to balance reducing equivalent allocation among detoxification, oxidative stress defense, and growth-related processes, ultimately affecting fermentation performance.

To survive under aldehyde stress, *S. cerevisiae* has evolved adaptive mechanisms involving detoxification pathways, oxidative stress defense, membrane remodeling, transporter-mediated efflux, and metabolic reprogramming. Among these regulatory systems, the transcription factor Yap1p plays a central role in coordinating antioxidant responses, glutathione metabolism, multidrug transport, and intracellular redox homeostasis. Several Yap1p-regulated transporters, including Atr1p and Flr1p, have been shown to contribute substantially to cellular tolerance toward aromatic aldehydes and other lignocellulose-derived inhibitors [16,17]. However, these studies have largely focused on individual protective pathways, whereas the global regulatory networks involved in syringaldehyde adaptation remain poorly characterized.

Transcriptomic studies have revealed extensive cellular reprogramming in response to furfural, 5-hydroxymethylfurfural, and selected lignin-derived aldehydes, involving pathways associated with redox metabolism, energy production, ribosome biogenesis, and stress signaling [18,19,20]. However, despite the recognized toxicity of syringaldehyde and its prevalence in lignocellulosic hydrolysates, the global transcriptional response and regulatory mechanisms underlying yeast adaptation to this inhibitor remain poorly understood. Existing studies have primarily described the inhibitory effects of syringaldehyde on growth and fermentation, with limited understanding of how physiological damage, metabolic remodeling, and transcriptional regulation are coordinated during cellular adaptation.

To address these knowledge gaps, the present study systematically investigated the physiological and transcriptomic responses of *S. cerevisiae* to syringaldehyde exposure. Fermentation performance, cellular integrity, and oxidative damage were evaluated to characterize the physiological consequences of inhibitor stress, while RNA sequencing (RNA-seq) was employed to elucidate the global transcriptional reprogramming associated with cellular adaptation. By integrating physiological, biochemical, and transcriptomic analyses, this study provides new insights into the mechanisms underlying syringaldehyde toxicity and yeast adaptation to aromatic aldehyde stress, while identifying potential molecular targets for improving yeast tolerance to lignocellulose-derived inhibitors.

## 2. Materials and Methods

### 2.1. Strains and Culture Conditions

The *Saccharomyces cerevisiae* GJ2008 strain was obtained from the Fermentation Engineering Research Institute of Guangxi University of Science and Technology. GJ2008 is an ethanol-fermenting strain with high-sugar tolerance and stable fermentation performance, and was selected as a fermentation strain to evaluate the physiological response to lignocellulose-derived aromatic aldehyde stress under high-sugar fermentation conditions. The strain was revived and pre-cultured in YPD medium (2% glucose, 2% peptone, and 1% yeast extract, natural pH) and cultivated at 30 °C and 150 rpm for 12 h to obtain a primary seed culture. The seed culture was subsequently transferred into fresh YPD medium at an inoculation rate of 10% (*v*/*v*) and cultivated to the logarithmic growth phase. The yeast cells were collected and adjusted to 1 × 10^8^ colony-forming units (CFU)/mL before centrifugation. After washing twice with sterile deionized water, the cells were resuspended in sterile deionized water to obtain a 10-fold concentrated yeast suspension (approximately 1 × 10^9^ CFU/mL), which was used for subsequent experiments.

### 2.2. Effects of Syringaldehyde on Growth of S. cerevisiae

Syringaldehyde (purity ≥ 98%) was purchased from Shanghai Macklin Biochemical Technology Co., Ltd. (Shanghai, China). The concentrated yeast suspension was inoculated into YPD medium containing different concentrations of syringaldehyde (0, 0.5, 1.0, 1.2, 1.4, 1.6, 1.8, and 2.0 g/L) at an inoculation rate of 1% (*v*/*v*). Syringaldehyde was dissolved in dimethyl sulfoxide (DMSO) before addition to the culture medium [21], and the final concentration of DMSO was maintained at 0.5% (*v*/*v*) in both control and syringaldehyde-treated groups to ensure identical solvent conditions. Cultivation was performed in 500 mL shake flasks containing 200 mL of culture medium at 30 °C and 150 rpm. During cultivation, samples were collected at regular intervals. Cell growth was monitored by measuring the optical density of the culture suspension at 560 nm (OD_560_) using sterile water as a blank control. Samples exceeding the linear detection range were appropriately diluted before measurement. Growth curves were constructed based on the OD_560_ values.

### 2.3. Ethanol Fermentation Assay

Based on the concentration-dependent growth inhibition observed in YPD medium, 1.4 g/L syringaldehyde was selected as a defined stress concentration for subsequent high-sugar fermentation experiments to evaluate its effects on fermentation performance. The concentrated yeast suspension was inoculated into sucrose fermentation medium (25% sucrose, 2% peptone, and 1% yeast extract) at an inoculation rate of 1% (*v*/*v*). Fermentation was conducted at 30 °C and 150 rpm for 36 h, with a control group lacking syringaldehyde and a treatment group supplemented with 1.4 g/L syringaldehyde. During fermentation, samples were collected at regular intervals. Concentrations of glucose, fructose, and sucrose were determined by high-performance liquid chromatography (HPLC) using an Agilent 1260 Infinity II system (Agilent Technologies, Santa Clara, CA, USA), and ethanol concentration was quantified by gas chromatography (GC) using an Agilent 8890 GC system (Agilent Technologies, Santa Clara, CA, USA), following the analytical procedures described previously [22].

### 2.4. Scanning Electron Microscopy (SEM)

The concentrated yeast suspension was inoculated into sucrose fermentation medium at an inoculation rate of 1% (*v*/*v*) and cultivated at 30 °C and 150 rpm until the logarithmic growth phase. Subsequently, syringaldehyde was added to achieve final concentrations of 0 g/L (control) and 1.4 g/L (treatment), and cultivation was continued for an additional 3 h before sample collection. Cells were harvested by centrifugation (6000× *g*, 10 min, 4 °C), washed three times with phosphate-buffered saline (PBS), and fixed overnight at 4 °C with an equal volume of 2.5% (*v*/*v*) glutaraldehyde. Sample preparation was performed according to the method described by Rozali et al. [23]. Following fixation, samples were dehydrated through a graded ethanol series (20%, 50%, 70%, 80%, 90%, and 100%). The samples were subsequently frozen at −80 °C and lyophilized under vacuum. Freeze-dried yeast cells were mounted on aluminium stubs, sputter-coated with gold, and observed using a scanning electron microscope (Phenom ProX Desktop SEM, Phenom-World, Eindhoven, The Netherlands).

### 2.5. Assessment of Extracellular Ultraviolet (UV)-Absorbing Components

Changes in extracellular UV-absorbing components were evaluated by measuring the absorbance of culture supernatants at 260 and 280 nm according to the method of Xiang et al. [24] with slight modifications. Yeast cells exposed to 1.4 g/L syringaldehyde were sampled after 0, 3, and 6 h of treatment. The collected culture samples were immediately heated in a boiling water bath for 5 min to terminate enzymatic activity and then centrifuged at 6000 r/min for 10 min. The supernatant was collected, and the absorbance values at 260 and 280 nm were measured to evaluate changes in extracellular UV-absorbing components.

### 2.6. Determination of TBARS Levels

Thiobarbituric acid reactive substances (TBARS) levels were determined using the thiobarbituric acid (TBA) method described by Ribeiro et al. [25] with slight modifications. Yeast cells treated with syringaldehyde for 0, 3, and 6 h were collected, washed with sterile water, and the cell biomass was determined by oven-drying the harvested cells to constant weight. The samples were then mixed with 10% (*w*/*v*) trichloroacetic acid (TCA) extraction solution and 0.6% (*w*/*v*) thiobarbituric acid (TBA) reagent. A 0.6% (*w*/*v*) TBA solution without cell samples was used as the blank control. After thorough mixing, the samples were heated in a boiling water bath for 15 min and then rapidly cooled. The mixtures were centrifuged at 8000 r/min for 5 min, and the absorbance of the supernatant was measured at 450, 532, and 600 nm. TBARS levels were calculated based on the standard curve and expressed as malondialdehyde (MDA) equivalents according to the method described by Ribeiro et al. [25].

### 2.7. Determination of Intracellular Glycerol Content

Yeast cells were cultivated and treated with syringaldehyde as described above, and samples were collected after 6 and 12 h of exposure. The cells were disrupted by ultrasonic treatment under ice-bath conditions (200 W, 3 s pulse, 10 s interval, repeated 30 times). The samples were centrifuged at 12,000 r/min and 4 °C for 10 min, and the supernatants were collected for glycerol determination. Intracellular glycerol content was measured according to the instructions provided with the glycerol assay kit (Glycerol Assay Kit, Quanzhou Ruixin Biological Technology Co., Ltd., Quanzhou, China).

### 2.8. Fourier Transform Infrared (FTIR) Spectroscopy

Yeast cells treated with syringaldehyde for 3 h were collected, washed with sterile water, and freeze-dried under vacuum. Approximately 2 mg of freeze-dried yeast biomass was thoroughly mixed with 200 mg of dry potassium bromide (KBr), ground uniformly, and compressed into transparent pellets. FTIR spectra obtained using an INVENIO R FTIR spectrometer (BRUKER OPTIK GmbH, Ettlingen, Germany) evaluated to assess biochemical alterations induced by syringaldehyde treatment according to the method of Favaro et al. [26].

### 2.9. RNA Extraction, Library Construction, and Transcriptome Sequencing

Yeast cells were cultivated to the logarithmic growth phase and treated with 1.4 g/L syringaldehyde for 2 h. Cells were harvested by centrifugation at 8000 r/min for 3 min, washed three times with sterile water, rapidly frozen in liquid nitrogen for 15 min, and stored at −80 °C until analysis. Total RNA was extracted according to the manufacturer’s instructions using a yeast RNA extraction kit. RNA integrity and potential genomic DNA contamination were evaluated by agarose gel electrophoresis, while RNA concentration and purity were determined using a NanoDrop 2000 spectrophotometer (Thermo Fisher Scientific, Wilmington, DE, USA).

Qualified RNA samples were subjected to mRNA enrichment using oligo(dT) magnetic beads and subsequently fragmented into short fragments in fragmentation buffer. First-strand cDNA was synthesized using mRNA as the template and random hexamer primers, followed by second-strand cDNA synthesis to generate double-stranded cDNA. The resulting cDNA underwent end repair, A-tailing, adapter ligation, and PCR amplification to construct sequencing libraries. Libraries that passed quality control were sequenced on an Illumina NovaSeq 6000 platform (Illumina, San Diego, CA, USA). Transcriptome sequencing was performed by Shanghai Majorbio Bio-pharm Technology Co., Ltd. (Shanghai, China).

### 2.10. Transcriptome Data Processing and Analysis

Raw sequencing data were converted into raw reads through base-calling analysis. Low-quality reads, adapter-contaminated reads, sequencing primer-contaminated reads, and reads containing more than 10% ambiguous bases (N) were removed to obtain high-quality clean reads. Clean reads were aligned to the *Saccharomyces cerevisiae* reference genome using HISAT2, and the resulting mapped reads were used for subsequent analyses. The quality of the alignment results was evaluated prior to downstream analysis.

Gene expression levels were quantified using RSEM (RNA-Seq by Expectation Maximization). Differentially expressed genes (DEGs) between treatment groups were identified using DESeq2 with thresholds of |log_2_FC| ≥ 1 and q-value < 0.05. Functional annotation of DEGs was performed using the Saccharomyces Genome Database (SGD). Gene Ontology (GO) enrichment analysis and Kyoto Encyclopedia of Genes and Genomes (KEGG) pathway enrichment analysis were conducted using the DAVID and KOBAS databases.

### 2.11. RT-qPCR Analysis

To examine the expression patterns of RNA-seq-derived DEGs, nine representative genes were selected for RT-qPCR analysis. *ACT1* was used as the internal reference gene for normalization. Gene-specific primers were designed using Primer Premier 5 software, and the primer sequences are listed in Table S1. Total RNA extracted as described above was reverse-transcribed into cDNA using the FastKing One-Step RT-PCR Kit (with gDNA removal) (Tiangen Biotech Co., Ltd., Beijing, China) according to the manufacturer’s instructions. Quantitative PCR reactions were prepared on ice in a total volume of 10 μL, containing 5 μL of 2× PowerUp SYBR Green Master Mix, 0.5 μL each of forward and reverse primers, 1 μL of cDNA template, and 3 μL of ddH_2_O. RT-qPCR was performed using a real-time PCR system under the following conditions: initial denaturation at 95 °C for 120 s; 40 cycles of denaturation at 95 °C for 15 s, annealing at 55–60 °C for 15 s, and extension at 72 °C for 60 s; followed by melt-curve analysis at 95 °C for 15 s, 60 °C for 60 s, and 95 °C for 15 s. Each sample was analyzed using three biological replicates. Relative gene expression levels were calculated using the 2^−ΔΔCt^ method, and the expression trends were compared with those obtained from transcriptomic sequencing.

### 2.12. Data Statistics and Analysis

All experiments were performed with at least three biological replicates, and the results are presented as mean ± standard deviation (SD). Statistical analyses were conducted using GraphPad Prism 8.1 software. Differences among groups were evaluated by one-way analysis of variance (ANOVA), and a *p*-value < 0.05 was considered statistically significant. All figures were generated using Origin 9.5 software.

## 3. Results

### 3.1. Effects of Syringaldehyde on Fermentation Performance

#### 3.1.1. Effects of Syringaldehyde on Cell Growth

**Figure 1:**
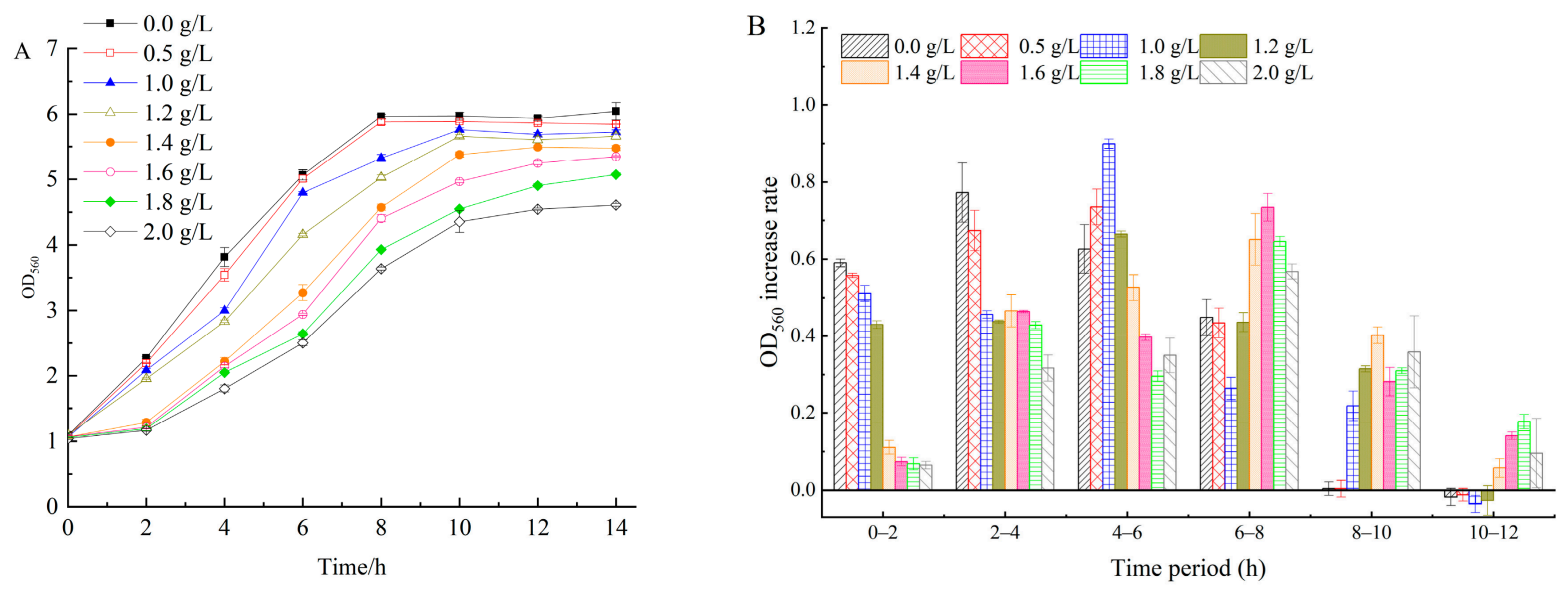
Effects of different concentrations of syringaldehyde on the growth of *Saccharomyces cerevisiae* GJ2008. (**A**) Growth curves of *S. cerevisiae* GJ2008 under different syringaldehyde concentrations. (**B**) Changes in OD_560_ increase rate during different growth periods under syringaldehyde stress.

To evaluate the inhibitory effect of syringaldehyde on yeast growth, *Saccharomyces cerevisiae* was cultivated in YPD medium supplemented with different concentrations of syringaldehyde (0–2.0 g/L), and growth curves were monitored (Figure 1). Syringaldehyde inhibited yeast growth in a concentration-dependent manner. At 0.5 g/L, the growth profile was comparable to that of the control, indicating that this concentration of syringaldehyde exerted little effect on cell proliferation. Increasing the syringaldehyde concentration to 1.0–1.2 g/L moderately prolonged the lag phase, whereas concentrations of 1.4–2.0 g/L caused stronger growth inhibition, characterized by delayed entry into the stationary phase and progressively reduced final OD_560_ values. At the highest concentration tested (2.0 g/L), the stationary phase was reached approximately 16 h after inoculation, compared with approximately 8 h in the control. Consistent with the growth curves, analysis of OD_560_ increase rates further demonstrated that syringaldehyde progressively delayed rapid growth and reduced growth dynamics in a concentration-dependent manner (Figure 1B).

Based on these results, 1.4 g/L syringaldehyde was selected as a representative stress concentration for subsequent high-sugar fermentation and physiological analyses because it caused a clear inhibitory effect on yeast growth while still maintaining sufficient cell growth for downstream characterization.

#### 3.1.2. Effects of Syringaldehyde on Sugar Utilization

**Figure 2:**
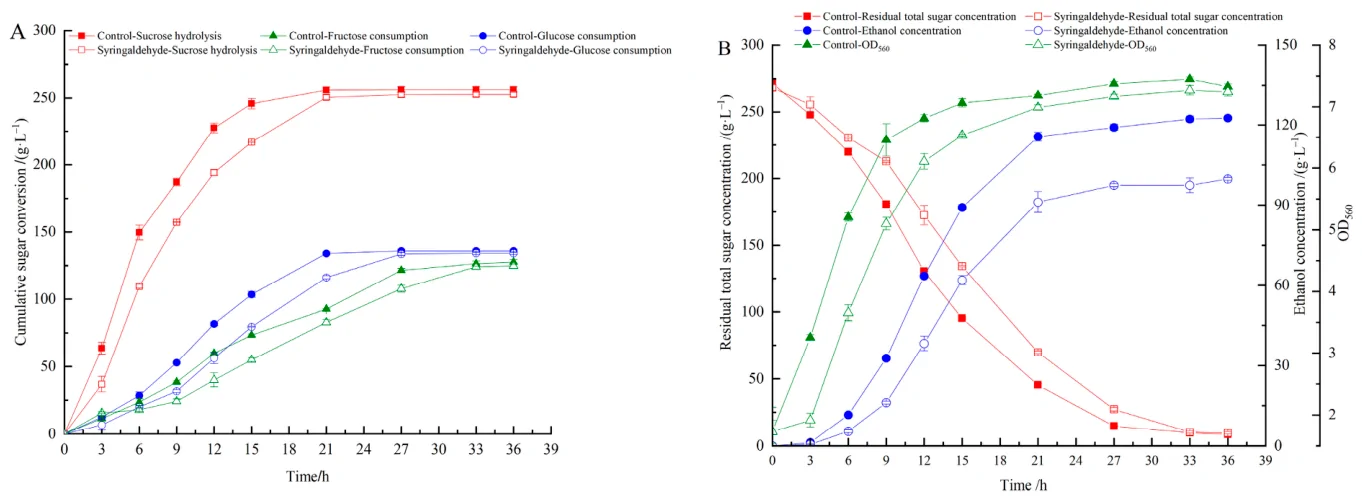
Fermentation profiles of *Saccharomyces cerevisiae* GJ2008 under syringaldehyde stress. (**A**) Sucrose hydrolysis and glucose/fructose consumption; (**B**) cell growth, residual sugar concentration, and ethanol production. Control represents the control group; Syringaldehyde, the treatment group supplemented with 1.4 g/L syringaldehyde.

To investigate the effect of syringaldehyde on sugar utilization and fermentation performance, *S. cerevisiae* was cultivated in medium containing 250 g/L sucrose with or without 1.4 g/L syringaldehyde. Changes in sucrose hydrolysis and glucose and fructose consumption are shown in Figure 2A. Although both the control and syringaldehyde-treated groups exhibited similar overall patterns of sucrose hydrolysis and monosaccharide utilization, syringaldehyde markedly delayed sugar consumption during the early stage of fermentation. In the control group, glucose and fructose concentrations began to decline rapidly after approximately 6 h and 9 h of fermentation, respectively, whereas these decreases were postponed to approximately 9 h and 12 h in the syringaldehyde-treated group, representing an approximately 3 h delay.

Accordingly, the rates of sucrose hydrolysis and glucose and fructose consumption were consistently lower in the treated group during the initial fermentation period (0–12 h). As fermentation proceeded, the differences between the two groups gradually diminished, and by 36 h glucose was almost completely consumed in both groups, whereas a small amount of fructose remained. These results indicate that syringaldehyde primarily affected the kinetics of sugar utilization rather than the final extent of sugar consumption, with the inhibitory effect being most evident during the early stage of ethanol fermentation.

#### 3.1.3. Effects of Syringaldehyde on Ethanol Fermentation

The effects of syringaldehyde on biomass accumulation, residual sugar concentration, and ethanol production are presented in Figure 2B, and the corresponding fermentation parameters are summarized in Table 1. During sucrose ethanol fermentation, syringaldehyde treatment exerted only a limited effect on final OD_560_ value, with the maximum optical density decreasing by 2.35% compared with the control. However, the early-stage fermentation process was clearly delayed. Consistent with the delayed glucose and fructose consumption shown in Figure 2A, the total sugar consumption rate in the syringaldehyde-treated group was lower than that of the control during the initial fermentation period (0–12 h). As fermentation proceeded, this difference gradually diminished, and no significant difference in residual total sugar concentration was observed between the two groups at 36 h.

Despite the relatively small changes in final OD_560_ value and residual sugar concentration, ethanol production was markedly impaired by syringaldehyde stress. The ethanol accumulation rate in the treated group was lower than that of the control, particularly during the early stage of fermentation. Although ethanol production partially recovered during the middle and late stages, the final ethanol concentration reached only 99.81 g/L, which was significantly lower than that of the control group (122.69 g/L), representing an 18.65% decrease. Correspondingly, total sugar fermentation efficiency, consumed-sugar fermentation efficiency, and sugar-to-ethanol conversion efficiency decreased by 17.64%, 16.60%, and 17.26%, respectively (Table 1).

Collectively, these results demonstrate that 1.4 g/L syringaldehyde had only a limited effect on final OD_560_ value and sugar consumption but markedly reduced ethanol production and sugar-to-ethanol conversion efficiency. The inhibitory effect of syringaldehyde on sucrose ethanol fermentation was therefore primarily reflected in delayed early-stage sugar utilization and reduced carbon conversion to ethanol.

### 3.2. Effects of Syringaldehyde on Cell Envelope Integrity and Oxidative Stress

#### 3.2.1. Cell Morphology and Cell-Envelope Alterations

**Figure 3:**
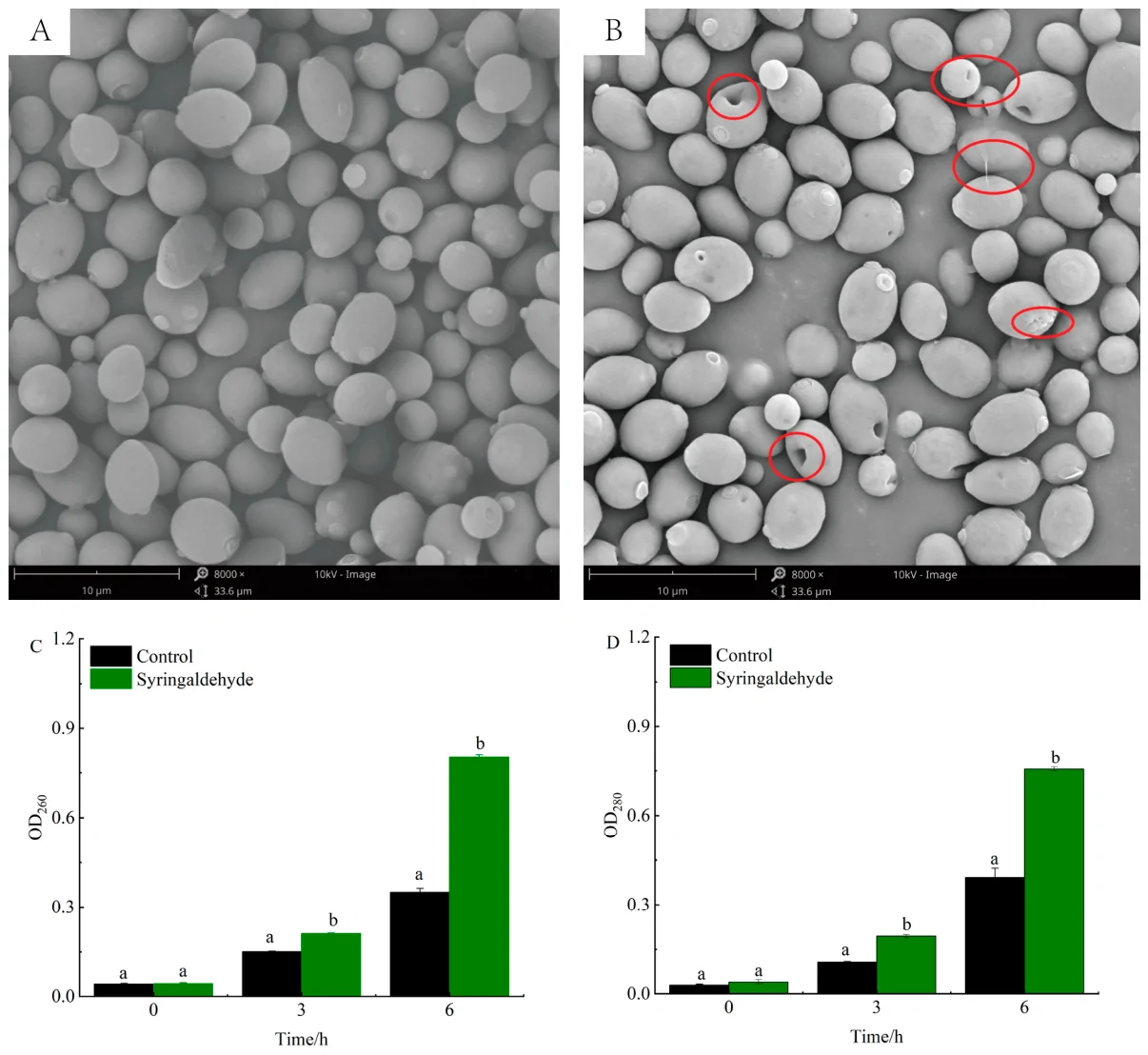
Cell-envelope alterations of *Saccharomyces cerevisiae* GJ2008 under syringaldehyde stress. (**A**,**B**) Scanning electron microscopy images of cells from the Control (**A**) and Syringaldehyde-treated (**B**) groups. Red circles indicate representative surface depressions and localized cavities in syringaldehyde-treated cells. (**C**,**D**) Changes in extracellular absorbance at 260 nm (**C**) and 280 nm (**D**) after syringaldehyde exposure. Different lowercase letters indicate statistically significant differences among groups (*p* < 0.05).

To investigate the effects of syringaldehyde on yeast cell-envelope structure, cell morphology was examined by scanning electron microscopy (SEM), and the release of intracellular components was analyzed by measuring extracellular UV-absorbing substances and proteins (Figure 3). SEM observations showed that cells in the control group exhibited a typical oval morphology with smooth surfaces, intact cell walls, and distinct bud scars. In contrast, cells exposed to 1.4 g/L syringaldehyde displayed obvious morphological abnormalities, including surface depressions, localized cavities, and cell aggregation, suggesting alterations in yeast cell-envelope structure under syringaldehyde stress.

The SEM observations were further supported by analysis of intracellular component release. Compared with the control group, the extracellular absorbance values at 260 and 280 nm increased significantly following syringaldehyde treatment (*p* < 0.05) and continued to increase with prolonged exposure. After 3 h of treatment, the extracellular absorbance at 260 and 280 nm increased to 1.40- and 1.80-fold of the control levels, respectively. After 6 h of exposure, these values increased to 2.29- and 1.93-fold of the control levels, respectively. The consistency of SEM observations and intracellular component release measurements provides complementary evidence that syringaldehyde induces structural and functional alterations of the yeast cell envelope, leading to increased release of intracellular components.

#### 3.2.2. Lipid Peroxidation and Biochemical Alterations

**Figure 4:**
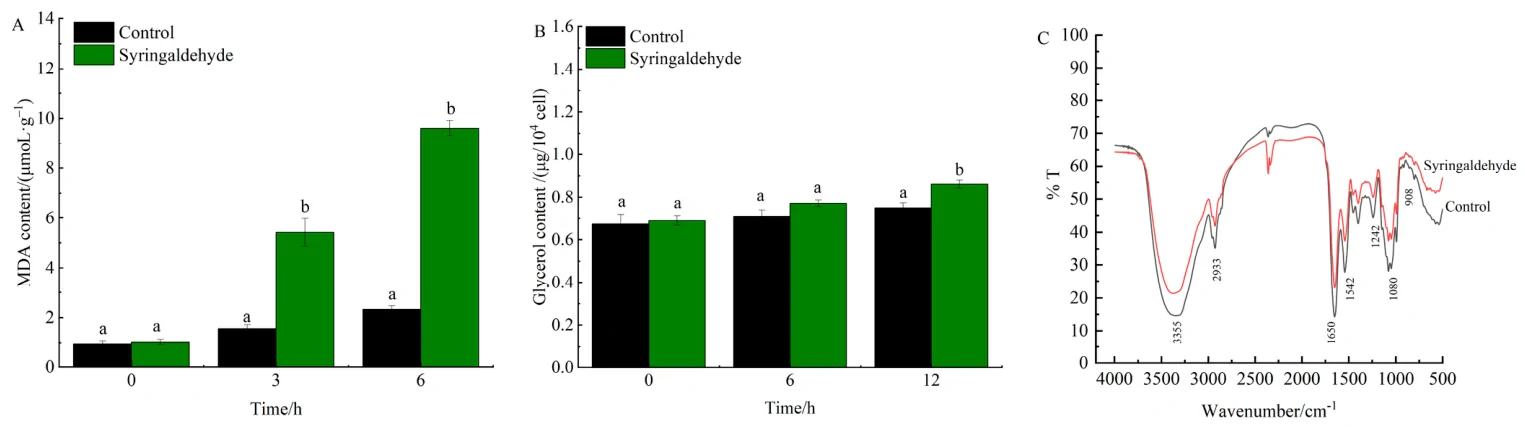
Biochemical characterization of *Saccharomyces cerevisiae* GJ2008 under syringaldehyde stress. (**A**) Intracellular MDA-equivalent levels determined by the TBA assay; (**B**) Intracellular glycerol content; (**C**) FTIR spectra of cells before and after syringaldehyde treatment. Different lowercase letters in panels (**A**,**B**) indicate statistically significant differences among groups (*p* < 0.05).

To further evaluate whether membrane damage was accompanied by oxidative injury, intracellular MDA-equivalent levels were determined using the TBA assay following syringaldehyde treatment, and biochemical alterations were analyzed by Fourier transform infrared (FTIR) spectroscopy (Figure 4). Compared with the control group, intracellular MDA-equivalent levels increased significantly following syringaldehyde treatment (*p* < 0.05) and continued to accumulate with increasing exposure time. After 3 h and 6 h of treatment, MDA-equivalent levels increased by 3.47-fold and 4.13-fold of the control levels, respectively, indicating enhanced membrane lipid peroxidation under syringaldehyde stress.

FTIR analysis further revealed noticeable shifts in several characteristic absorption bands following syringaldehyde treatment. The broad absorption band at 3355 cm^−1^ shifted to 3371 cm^−1^, while the band at 2928 cm^−1^ shifted to 2933 cm^−1^. Moreover, alterations were observed in the amide I, amide II, and polysaccharide-associated regions, indicating changes in membrane proteins, membrane lipids, and cell wall polysaccharides. No additional absorption peaks were detected, suggesting that these changes primarily reflected alterations in the structural organization of existing cellular components rather than major changes in the overall biochemical composition of the cells. Taken together, the increased MDA-equivalent levels and FTIR spectral changes provide complementary evidence that syringaldehyde induces lipid peroxidation-associated oxidative stress accompanied by structural alterations in cell-envelope components, consistent with the cellular structural changes observed by SEM and extracellular absorbance analyses.

#### 3.2.3. Intracellular Glycerol Accumulation

Intracellular glycerol accumulation was determined to evaluate the osmotic adaptive response of *S. cerevisiae* to syringaldehyde stress (Figure 4B). Compared with the control group, intracellular glycerol content showed no significant difference at 0 h. After 6 h of syringaldehyde treatment, glycerol content showed an increasing trend, but the difference was not statistically significant (*p* > 0.05). After 12 h of treatment, intracellular glycerol content was significantly higher than that in the corresponding control group (*p* < 0.05), increasing by approximately 14.70%. These findings indicate that glycerol accumulation occurred primarily during prolonged syringaldehyde exposure, representing a delayed adaptive response rather than an immediate protective mechanism.

### 3.3. Transcriptomic Analysis of the Response to Syringaldehyde Stress

#### 3.3.1. Global Transcriptional Response to Syringaldehyde Stress

To elucidate the molecular response of *S. cerevisiae* to syringaldehyde stress, transcriptome sequencing was performed using cells exposed to 1.4 g/L syringaldehyde for 2 h. High-quality sequencing data were obtained for all samples, with more than 46.79 million clean reads generated for each library. The average Q20 and Q30 values exceeded 98% and 94%, respectively, while more than 95% of the clean reads were successfully mapped to the reference genome, indicating that the sequencing data were of sufficient quality for downstream analyses.

Differential expression analysis identified 496 differentially expressed genes (DEGs) between the syringaldehyde-treated and control groups, including 107 upregulated genes and 389 downregulated genes (Figure S1). The predominance of downregulated genes indicates that transcriptional repression represented the dominant early response of *S. cerevisiae* to syringaldehyde stress.

#### 3.3.2. Functional Enrichment Analysis of Differentially Expressed Genes

**Figure 5:**
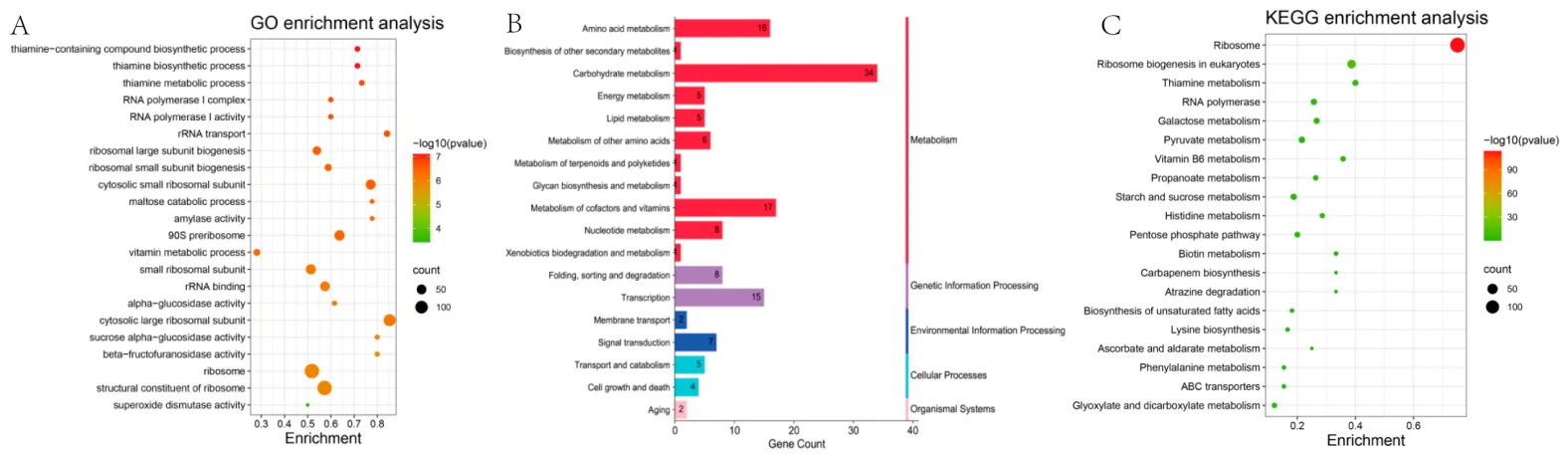
Functional enrichment analysis of differentially expressed genes (DEGs) under syringaldehyde stress. (**A**) GO enrichment analysis; (**B**) KEGG pathway classification statistics; (**C**) KEGG pathway enrichment analysis.

To further characterize the biological functions affected by syringaldehyde stress, Gene Ontology (GO) and Kyoto Encyclopedia of Genes and Genomes (KEGG) enrichment analyses were performed using the identified DEGs (Figure 5). GO enrichment analysis revealed that the DEGs were predominantly associated with ribosome assembly, ribosome biogenesis, thiamine metabolism, vitamin metabolism, RNA polymerase I complex, α-glucosidase activity, and superoxide dismutase activity, indicating that syringaldehyde markedly affected protein synthesis, cofactor metabolism, and oxidative stress-related processes.

Consistent with the GO analysis, KEGG enrichment analysis identified significant enrichment of pathways involved in ribosome, ribosome biogenesis, thiamine metabolism, RNA polymerase, galactose metabolism, pyruvate metabolism, starch and sucrose metabolism, pentose phosphate pathway, unsaturated fatty acid biosynthesis, ABC transporters, and glyoxylate metabolism. Together, the GO and KEGG enrichment analyses consistently identified ribosome biogenesis, cofactor metabolism, and central carbon metabolism as the major biological processes affected by syringaldehyde stress.

#### 3.3.3. Expression Profiles of Representative Differentially Expressed Genes

To further elucidate the molecular basis of the adaptive response to syringaldehyde stress, representative DEGs involved in the major enriched pathways were analyzed in detail (Table 2).

Ribosome biogenesis and protein synthesis: Ribosome biogenesis was one of the most strongly affected cellular processes. Numerous genes encoding ribosomal structural proteins were significantly downregulated, including *RPS22A*, *RPS26B*, *RPS20*, *RPS9B*, *RPS12*, *RPL9A*, *RPL26B*, *RPL30*, *RPL5*, *RPL11A*, *RPL36B*, *RPL31B*, *RPL8B*, *RPL24A*, and *RPL3*. In addition, several genes involved in ribosome assembly and rRNA maturation, including *NOP56*, *NOP58*, *NOG2*, *NOP4*, *EMG1*, and *MDN1*, also showed significant downregulation. These results indicate that syringaldehyde broadly suppresses ribosome biogenesis and protein synthesis.

Cofactor biosynthesis: Genes involved in cofactor biosynthesis were also coordinately downregulated. Multiple genes associated with thiamine biosynthesis (*THI5*, *THI11*, *THI12*, *THI13*, *THI21*, *THI22*, and *THI80*) exhibited significantly decreased expression. Likewise, genes involved in vitamin metabolism (*PHO3*, *BUD16*, *SNO2*, *SNO3*, *SNZ2*, and *SNZ3*) and biotin biosynthesis (*BIO2* and *BIO4*) were also downregulated, suggesting that syringaldehyde markedly affects cofactor metabolism.

Carbon utilization, pentose phosphate pathway, and aldehyde detoxification: In contrast, genes involved in carbon utilization, pentose phosphate metabolism, and aromatic aldehyde detoxification were selectively upregulated. Genes involved in sugar utilization, including *SUC2* and members of the *IMA* family (*IMA1*–*IMA5*), exhibited increased expression. Several pentose phosphate pathway genes, including *SOL4*, *GND2*, and *TKL2*, were also significantly upregulated. Among all upregulated genes, *ADH7* exhibited the highest induction level (log_2_FC = 5.25). Meanwhile, *ALD3* and *ACS1* were also upregulated, whereas *ALD5* and *PDC5* were downregulated.

Overall, syringaldehyde stress was characterized by coordinated repression of genes involved in ribosome biogenesis and cofactor biosynthesis, accompanied by selective induction of genes associated with carbon utilization, the pentose phosphate pathway, and aromatic aldehyde detoxification.

#### 3.3.4. RT-qPCR Analysis of Selected DGEs

To examine the expression patterns of RNA-seq-derived DEGs, nine representative genes were selected for RT-qPCR analysis. As shown in Figure S2, the expression trends determined by RT-qPCR were generally consistent with those obtained from transcriptome sequencing, providing additional support for the expression patterns identified by RNA-seq.

## 4. Discussion

Syringaldehyde markedly impaired ethanol fermentation by *S. cerevisiae*, yet its inhibitory mechanism remains much less understood than those of other lignocellulose-derived aldehydes, such as furfural, 5-hydroxymethylfurfural (HMF), and vanillin. The physiological and transcriptomic analyses presented here indicate that syringaldehyde induces cell-envelope alterations, oxidative imbalance, and metabolic dysregulation. Together, these interconnected responses reveal that syringaldehyde stress involves both physiological disturbance and adaptive metabolic adjustment, providing a mechanistic framework for understanding how yeast responds to lignin-derived aromatic aldehyde stress.

Under the selected stress condition of 1.4 g/L, syringaldehyde had only a minor effect on final OD_560_ value but markedly reduced ethanol production and sugar-to-ethanol conversion efficiency. Sucrose hydrolysis and glucose/fructose utilization were delayed during early fermentation, yet the final residual sugar concentration remained comparable to that of the control, suggesting that syringaldehyde primarily impaired fermentation efficiency rather than sugar utilization capacity. Previous studies have shown that detoxification of lignocellulose-derived aldehydes imposes an additional metabolic burden on *S. cerevisiae* by consuming reducing equivalents and disturbing intracellular redox homeostasis, ultimately compromising ethanol production [10,18]. Consistent with this view, although most fermentable sugars were eventually consumed in the present study, the lower ethanol production suggests that a greater proportion of cellular resources may have been redirected toward cellular maintenance and stress adaptation processes, potentially limiting ethanol biosynthesis.

Cell-envelope alterations represent one of the physiological responses of *S. cerevisiae* to syringaldehyde stress. SEM analysis revealed morphological abnormalities, including surface depressions and localized cavities. These structural changes were accompanied by increased extracellular absorbance at 260 and 280 nm, suggesting enhanced release of intracellular UV-absorbing components and altered cell-envelope barrier properties under syringaldehyde stress. FTIR analysis further showed alterations in spectral regions associated with membrane lipids, proteins, and cell wall polysaccharides, indicating alterations in the structural organization and molecular components of the cell envelope. Taken together, these independent observations provide complementary evidence that syringaldehyde induces structural and functional alterations of the yeast cell envelope. Aromatic aldehydes have been reported to disturb membrane organization and associated transport processes, affecting membrane barrier properties and intracellular homeostasis [10]. These alterations may contribute to cellular stress by affecting membrane-associated physiological processes and increasing the burden of stress adaptation, thereby potentially contributing to delayed sugar utilization and the overall reduction in fermentation efficiency under syringaldehyde stress.

Oxidative damage was closely associated with the cell-envelope alterations observed under syringaldehyde stress. Intracellular MDA-equivalent levels increased markedly after syringaldehyde treatment and continued to rise throughout the exposure period, indicating progressive lipid peroxidation [27,28]. Increases in MDA-equivalent levels, which are commonly used as an indicator of lipid peroxidation, have also been documented in *S. cerevisiae* exposed to benzoic acid [29]. Together with previous reports describing oxidative damage caused by lignin-derived phenolic inhibitors [10], these observations suggest that lipid peroxidation represents a recurring feature of yeast responses to aromatic inhibitors rather than a unique consequence of syringaldehyde exposure. Because biological membranes are highly susceptible to ROS-mediated damage, oxidative stress may contribute to the cell-envelope alterations and membrane-associated changes observed during syringaldehyde exposure. This oxidative amplification may partly contribute to the reduced cellular functionality observed under syringaldehyde stress, as reflected by the decline in fermentation performance.

In response to syringaldehyde-induced stress and oxidative imbalance, *S. cerevisiae* gradually activated glycerol accumulation as a secondary adaptive response. Intracellular glycerol content changed only slightly during the early stage of syringaldehyde exposure but increased significantly after 12 h. This delayed accumulation indicates that glycerol biosynthesis was activated only after substantial cellular damage had already occurred, rather than serving as an immediate protective response. Glycerol is a well-established compatible solute that contributes to osmotic adjustment and stress tolerance in yeast [30,31]. Therefore, this response most likely functioned to mitigate the physiological consequences of syringaldehyde stress rather than to prevent the initial development of cellular damage. Consequently, although glycerol accumulation may have contributed to maintaining cellular viability, this delayed adaptive response was insufficient to restore fermentative capacity, highlighting the distinction between cellular survival and fermentative productivity under inhibitor exposure.

The combined burden of cellular stress and oxidative imbalance was accompanied by extensive transcriptional reprogramming, reflecting the adaptive response of *S. cerevisiae* to syringaldehyde exposure. One of the most prominent responses was the coordinated repression of genes involved in ribosome biogenesis and protein synthesis. Ribosome assembly is among the most energy-demanding anabolic processes in rapidly growing yeast cells, and coordinated downregulation of ribosomal protein genes (*RPL* and *RPS* families) together with ribosome biogenesis factors (e.g., *NOP* family members) is a well-established feature of the Environmental Stress Response (ESR). This transcriptional pattern indicates that *S. cerevisiae* reduced its investment in energy-intensive biosynthetic processes and redirected resources toward stress adaptation under syringaldehyde exposure. Because transcriptomic analysis was performed at a single sampling time point, these transcriptional changes most likely represent an early adaptive response rather than the overall physiological state throughout fermentation. Nevertheless, transient repression of ribosome biogenesis is expected to conserve cellular resources and reduce biosynthetic demand during the initial phase of stress adaptation, thereby facilitating survival under adverse conditions. Similar repression of ribosome-related processes has been observed under various environmental and inhibitor stresses, suggesting that reduced investment in biosynthetic activity may contribute to cellular adaptation during stress exposure. Beyond this general stress response, transcriptional changes affecting central metabolism and fermentation-associated pathways may provide additional insights into the reduced ethanol production observed under syringaldehyde stress. These alterations suggest a shift in cellular priorities from biosynthetic growth toward stress adaptation and detoxification.

Beyond the repression of ribosome biogenesis, syringaldehyde also coordinately suppressed genes involved in thiamine and biotin biosynthesis. Both vitamins serve as essential cofactors in yeast, supporting central carbon metabolism, ethanol fermentation, and cellular homeostasis [32]. Among these, the repression of thiamine biosynthesis was particularly notable. Thiamine pyrophosphate (TPP), the active form of thiamine, serves as an essential cofactor for pyruvate decarboxylase and several other enzymes involved in central metabolism [33]. The widespread downregulation of *THI* genes therefore suggests a reduced cellular capacity for TPP biosynthesis under syringaldehyde stress. Consistent with the altered regulation of thiamine metabolism, several genes associated with pyruvate decarboxylation showed differential expression, including downregulation of *PDC5*, which encodes a pyruvate decarboxylase isoform, whereas *PDC1* was upregulated. This pattern suggests that syringaldehyde stress may induce isoform-specific regulation of pyruvate decarboxylase rather than a simple suppression of pyruvate decarboxylation capacity. Although intracellular TPP levels and pyruvate decarboxylase activity were not determined in the present study, the altered expression of pyruvate decarboxylase-related genes, together with reduced thiamine biosynthetic capacity, suggests that pyruvate metabolism may be disturbed and potentially contribute to the decreased ethanol production observed under syringaldehyde stress. In addition, biotin serves as an essential cofactor for carboxylases involved in lipid metabolism and broader metabolic homeostasis. Suppression of biotin biosynthesis may further affect cellular metabolic homeostasis, although its specific contribution to cell-envelope alterations requires further investigation. Together with the repression of ribosome biogenesis discussed above, these findings indicate that cellular metabolism was broadly reprogrammed to prioritize stress adaptation at the expense of fermentative efficiency.

Counterbalancing the coordinated repression of biosynthetic pathways, genes associated with redox regulation and aromatic aldehyde detoxification were selectively induced under syringaldehyde stress. Notably, several genes associated with NADPH generation and central carbon metabolism, including *GND2*, *TKL2*, *SOL4*, and *NQM1*, were significantly upregulated, suggesting an increased demand for redox balancing and metabolic adjustment to support antioxidant defense and aldehyde detoxification. This response represents a conserved protective strategy previously reported for lignocellulose-derived aldehyde stress [10,18]. Because activation of the pentose phosphate pathway may redivert glucose-derived carbon away from glycolytic metabolism, its activation may indicate a metabolic redistribution in which cellular resources are preferentially allocated toward redox maintenance and inhibitor detoxification rather than maximal ethanol formation. Among the most responsive genes, *ADH7* showed exceptionally strong induction, and together with *ALD3* and *ACS1*, this points to the activation of aldehyde detoxification pathways. Although alcohol dehydrogenases participate in both ethanol metabolism and aldehyde reduction reactions, the strong induction of *ADH7* in this study is more likely associated with aldehyde detoxification under syringaldehyde stress rather than enhanced ethanol production. Together with the induction of PPP-related genes, the upregulation of *ADH7* suggests that reducing equivalents were preferentially utilized for detoxification processes under inhibitor stress. When considered together, the coordinated repression of biosynthetic pathways and induction of detoxification-related pathways suggest a metabolic trade-off during adaptation to syringaldehyde stress. Rather than sustaining maximal fermentative activity, yeast cells appear to have reallocated metabolic capacity toward maintaining redox homeostasis and detoxification. Although this adaptive response may enhance survival under inhibitor stress, it may simultaneously constrain resources available for efficient ethanol biosynthesis, thereby contributing to the reduced fermentation efficiency observed under syringaldehyde exposure.

**Figure 6:**
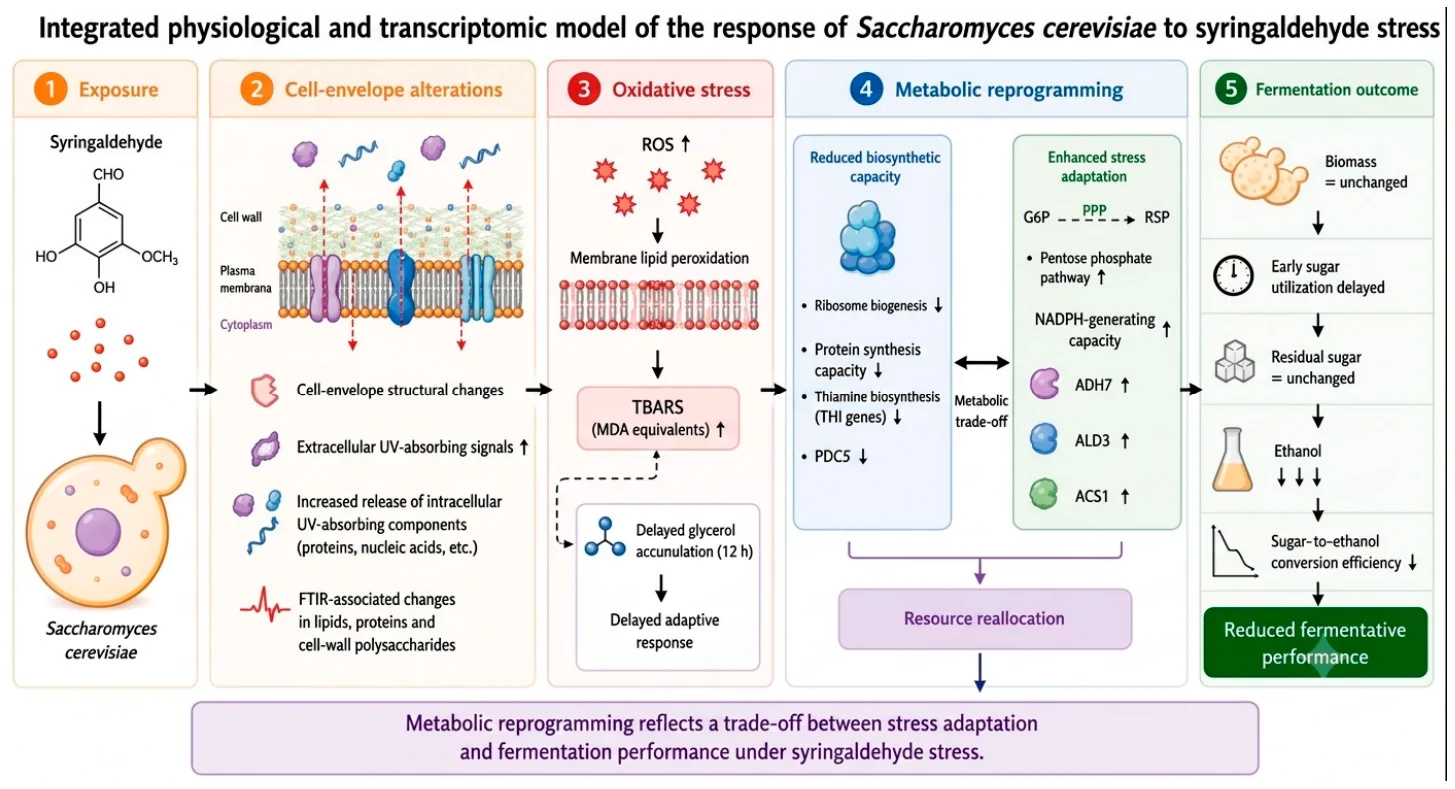
Proposed physiological and transcriptomic response of *Saccharomyces cerevisiae* to syringaldehyde stress. Arrows indicate the direction of the proposed responses or changes; ↑ and ↓ indicate increases and decreases, respectively.

Collectively, the physiological and transcriptomic evidence obtained in this study supports an integrated model of the *S. cerevisiae* response to syringaldehyde stress (Figure 6). Syringaldehyde exposure triggered coordinated physiological and transcriptional responses, including cell-envelope alterations, oxidative imbalance, and extensive metabolic reprogramming involving ribosome biogenesis, cofactor metabolism, pyruvate metabolism, and NADPH-dependent detoxification pathways. Rather than representing independent stress responses, these coordinated physiological and transcriptional changes collectively suggest a metabolic trade-off in which fermentative metabolism was reduced while cellular functions required for stress adaptation and detoxification were preferentially maintained. Importantly, this metabolic redistribution provides a potential explanation for the disproportionate reduction in ethanol production compared with biomass accumulation observed during fermentation. The differential regulation of fermentation-associated genes, including pyruvate decarboxylase-related genes and aldehyde detoxification genes, further suggests that altered carbon and redox metabolism may contribute to the reduced ethanol production phenotype. Although the activities of key enzymes involved in pyruvate metabolism and ethanol fermentation were not directly determined in the present study, future investigations of pyruvate decarboxylase and alcohol dehydrogenase activities will further clarify their roles in syringaldehyde-induced fermentation inhibition. Several responses identified here, including oxidative stress, pentose phosphate pathway induction, altered cofactor metabolism, and repression of ribosome biogenesis, are broadly consistent with previous studies on lignocellulose-derived aldehydes. Unlike previous studies, which have primarily emphasized intracellular redox imbalance and detoxification, the present study further highlights the contribution of cell-envelope alterations, cofactor metabolism, and transcriptional regulation of fermentation-associated pathways to syringaldehyde adaptation. These findings suggest that syringaldehyde elicits a coordinated stress response involving both cellular protection and metabolic adjustment rather than acting through a single dominant inhibitory mechanism. Therefore, the reduced ethanol production observed under syringaldehyde stress likely results from a redistribution of cellular resources away from fermentative productivity toward stress tolerance and detoxification.

From a practical standpoint, the complexity of syringaldehyde toxicity indicates that improving syringaldehyde tolerance may require coordinated optimization of multiple cellular processes rather than targeting a single pathway. Given that this inhibitor affects multiple cellular processes, including cell-envelope properties, cofactor metabolism, redox balance, and aldehyde detoxification capacity, effective strain improvement may require coordinated enhancement of these cellular processes. Strategies that concurrently maintain cellular homeostasis, improve detoxification efficiency, sustain cofactor availability, and preserve fermentative capacity may be more effective in maintaining ethanol productivity in the presence of lignin-derived aromatic inhibitors. Such strategies may contribute to the development of more robust industrial *S. cerevisiae* strains for lignocellulosic bioethanol production. The mechanistic framework proposed here therefore provides a conceptual basis for developing strategies to improve yeast tolerance to lignin-derived aromatic inhibitors.

## 5. Conclusions

This study systematically investigated the physiological and molecular responses of *Saccharomyces cerevisiae* to syringaldehyde stress by integrating fermentation analysis, cellular characterization, and transcriptomic profiling. Syringaldehyde markedly impaired ethanol fermentation while causing only a modest reduction in biomass accumulation. Physiological analyses revealed cell-envelope alterations associated with progressive lipid peroxidation, whereas transcriptomic analysis demonstrated coordinated repression of ribosome biogenesis and cofactor biosynthesis, together with transcriptional changes associated with cofactor metabolism, pyruvate metabolism, the pentose phosphate pathway, and aromatic aldehyde detoxification.

Together, these findings indicate that syringaldehyde inhibits ethanol fermentation through coordinated physiological alterations and metabolic reprogramming rather than through a single cellular target. The yeast response involved a trade-off between stress adaptation and fermentative productivity, characterized by reduced investment in biosynthetic processes, redistribution of metabolic resources toward redox maintenance and aldehyde detoxification, and altered regulation of fermentation-associated pathways. More broadly, this study advances understanding of how *S. cerevisiae* adapts to lignin-derived aromatic aldehyde stress and highlights the importance of coordinated regulation of cell-envelope properties, cofactor metabolism, fermentation-associated metabolism, and redox homeostasis in determining yeast robustness during lignocellulosic bioethanol production.

## Figures and Tables

**Table 1:** Fermentation performance of *Saccharomyces cerevisiae* GJ2008 under control and syringaldehyde stress conditions.

Amount of Syringaldehyde Added (g/L)	Initial Total Sugar (g/L)	Residual Total Sugar (g/L)	Residual Sucrose (g/L)	Residual Fructose (g/L)	Residual Glucose (g/L)	Final Ethanol Concentration (g/L)	Glucose Utilization Rate (%)	Fructose Utilization Rate (%)	Total Sugar Utilization Rate (%)	Total Sugar Fermentation Efficiency (%)	Consumption Sugar Fermentation Efficiency (%)	Sugar-to-Ethanol Conversion Rate (%)
0	271.62 ± 2.29 ^a^	8.54 ± 0.70 ^a^	0 ± 0.00 ^a^	8.54 ± 0.70 ^a^	0 ± 0.00 ^a^	122.69 ± 0.56 ^a^	100 ± 0.00 ^a^	93.67 ± 0.57 ^a^	96.86 ± 0.28 ^a^	88.40 ± 0.34 ^a^	90.54 ± 0.41 ^a^	46.64 ± 0.32 ^a^
1.4	268.26 ± 2.14 ^a^	9.57 ± 0.81 ^a^	0 ± 0.00 ^a^	9.57 ± 0.81 ^a^	0 ± 0.00 ^a^	99.81 ± 0.56 ^b^	100 ± 0.00 ^a^	92.80 ± 0.66 ^a^	96.43 ± 0.33 ^a^	72.81 ± 0.99 ^b^	75.51 ± 1.28 ^b^	38.59 ± 0.65 ^b^

^a,b^ Different lowercase letters indicate significant differences between groups (*p* < 0.001).

**Table 2:** Representative differentially expressed genes involved in stress response, cofactor metabolism, and fermentation-associated pathways under syringaldehyde stress.

Gene ID	Gene Name	Log_2_ FC	Gene Description
Differentially expressed genes related to ribosomes and their biosynthesis			
YGL147C	*RPL9A*	−3.85	60S ribosomal subunit protein
YJL190C	*RPS22A*	−3.77	40S ribosomal subunit protein
YER131W	*RPS26B*	−3.74	40S ribosomal subunit protein
YGR034W	*RPL26B*	−3.44	60S ribosomal subunit protein
YGL030W	*RPL30*	−3.26	60S ribosomal subunit protein
YPL131W	*RPL5*	−3.14	60S ribosomal subunit protein
YPR102C	*RPL11A*	−3.13	60S ribosomal subunit protein
YHL015W	*RPS20*	−2.96	40S ribosomal subunit protein
YPL249C-A	*RPL36B*	−2.91	60S ribosomal subunit protein
YLR406C	*RPL31B*	−2.86	60S ribosomal subunit protein
YLL045C	*RPL8B*	−2.81	60S ribosomal subunit protein
YBR189W	*RPS9B*	−2.79	40S ribosomal subunit protein
YOR369C	*RPS12*	−2.74	40S ribosomal subunit protein
YGL031C	*RPL24A*	−2.61	60S ribosomal subunit protein
YOR063W	*RPL3*	−2.60	60S ribosomal subunit protein
YNL178W	*RPS3*	−2.54	40S ribosomal subunit protein
YOL039W	*RPP2A*	−2.41	Ribosomal protein P2α, involved in the interaction between translation elongation factors and ribosomes
YLR167W	*RPS31*	−2.21	The fusion protein cleavage produces ribosomal protein S31 and ubiquitin, which can promote the assembly of ribosomal proteins into ribosomes.
YDL130W	*RPP1B*	−2.09	Ribosomal protein P1β
YLR340W	*RPP0*	−2.02	Ribosomal protein P0
YDR382W	*RPP2B*	−1.93	Ribosomal protein P2β
YLR197W	*NOP56*	−2.36	Nucleolar protein
YLR106C	*MDN1*	−1.14	Acts on ribosome biogenesis factors during 60S pre-assembly
YNR053C	*NOG2*	−2.23	Associated with the 60S ribosomal subunit in the nucleolus, essential for its nuclear export and maturation.
YPL043W	*NOP4*	−1.37	A nucleolar protein that is crucial for the processing, maturation of 27S pre-rRNA, and biogenesis of the large ribosomal subunit.
YLR186W	*EMG1*	−1.28	rRNA methyltransferase
YOR310C	*NOP58*	−2.02	Proteins involved in the production of mature rRNA and snoRNA, participating in pre-rRNA processing, 18S rRNA synthesis, and nucleolar small RNA synthesis
Differentially expressed genes related to cofactor metabolism			
YNL332W	*THI12*	−4.96	Proteins involved in the synthesis of thiamine precursor HMP
YJR156C	*THI11*	−3.84	Proteins involved in the synthesis of thiamine precursor HMP
YDL244W	*THI13*	−3.73	Proteins involved in the synthesis of thiamine precursor HMP
YFL058W	*THI5*	−3.64	Proteins involved in the synthesis of thiamine precursor HMP
YPR121W	*THI22*	−2.99	Protein similar to hydroxymethylpyrimidine phosphate kinase
YBR092C	*PHO3*	−1.82	Acidic phosphatase similar to Pho5p, hydrolyzes thiamine phosphate in the surrounding interstitial space, increasing cellular uptake of thiamine
YOR143C	*THI80*	−1.23	Thiamine pyrophosphate kinase
YPL258C	*THI21*	−1.17	Hydroxymethylpyrimidine (HMP) and HMP kinase, involved in thiamine biosynthesis
YEL029C	*BUD16*	−1.43	Putative pyridoxal kinase, a key enzyme involved in the synthesis of pyridoxal 5′-phosphate, the active form of vitamin B6
YFL060C	*SNO3*	−1.92	Induced in the absence of thiamine
YNL333W	*SNZ2*	−2.59	Pyridoxal phosphate biosynthesis protein
YNL334C	*SNO2*	−1.71	Induction in the absence of thiamine
YFL059W	*SNZ3*	−2.59	Pyridoxal phosphate biosynthesis protein
YGR286C	*BIO2*	−1.04	Biotin synthase, catalyzes the conversion of desulfurized biotin to biotin
YNR057C	*BIO4*	−1.46	Desulfurization biotin synthetase
Differentially expressed genes related to fermentation-associated metabolism and aldehyde detoxification			
YCR105W	*ADH7*	5.25	Alcohol dehydrogenase
YBR117C	*TKL2*	2.71	Transketolase
YGR256W	*GND2*	2.39	6-phosphogluconate dehydrogenase
YGR248W	*SOL4*	1.15	6-phosphogluconolactonase
YGR043C	*NQM1*	1.58	Transaldolase-like protein
YMR169C	*ALD3*	2.87	Cytosolic Aldehyde dehydrogenase
YAL054C	*ACS1*	1.44	Acetyl-CoA synthetase isoform
YLR134W	*PDC5*	−1.76	Pyruvate decarboxylase isoform
YLR044C	*PDC* *1*	1.33	Major pyruvate decarboxylase isozyme
YER073W	*ALD5*	−1.36	Mitochondrial aldehyde dehydrogenase
YGR287C	*IMA1*	1.01	Isomaltase
YOL157C	*IMA2*	1.98	Isomaltase
YIL172C	*IMA3*	1.91	Isomaltase
YJL221C	*IMA4*	1.91	Isomaltase
YJL216C	*IMA5*	1.04	Isomaltase
YIL162W	*SUC2*	1.86	Sucrose hydrolase
Differentially expressed genes related to cell wall biosynthesis			
YGR032W	*GSC2*	1.27	Catalytic subunit of 1,3-β-glucan synthase

## Data Availability

The original data presented in this study are openly available in the article. The raw transcriptomic sequencing data generated in this study have been deposited in the NCBI Sequence Read Archive (SRA) under BioProject accession number PRJNA1507869. Further inquiries can be directed to the corresponding author.

## Supplementary Materials

The following supporting information can be downloaded at: https://www.mdpi.com/article/10.3390/biology15161410/s1, Figure S1: Volcano plot of differentially expressed genes under syringaldehyde stress; Figure S2: Comparison of RT-qPCR and RNA-seq expression patterns of selected differentially expressed genes; Table S1: RT-qPCR Primer Sequences.

## Abbreviations

The following abbreviations are used in this manuscript:

DMSO	Dimethyl sulfoxide
SEM	Scanning electron microscopy
MDA	Malondialdehyde
TBA	Thiobarbituric acid
TCA	Trichloroacetic acid
FTIR	Fourier transform infrared
DEGs	Differentially expressed genes
SGD	Saccharomyces Genome Database
GO	Gene Ontology
KEGG	Kyoto Encyclopedia of Genes and Genomes
