# Supplementary material for: Integrated Physiological and Transcriptomic Analyses of Saccharomyces cerevisiae Under Syringaldehyde Stress
Source: Biology (Basel). 2026 Aug 17;15(16):1410. doi: 10.3390/biology15161410 (PMC13509397; doi:10.3390/biology15161410)
Supplement: Supplementary file 1 [file biology-15-01410-s001.zip › biology-4477588-supplementary.pdf]

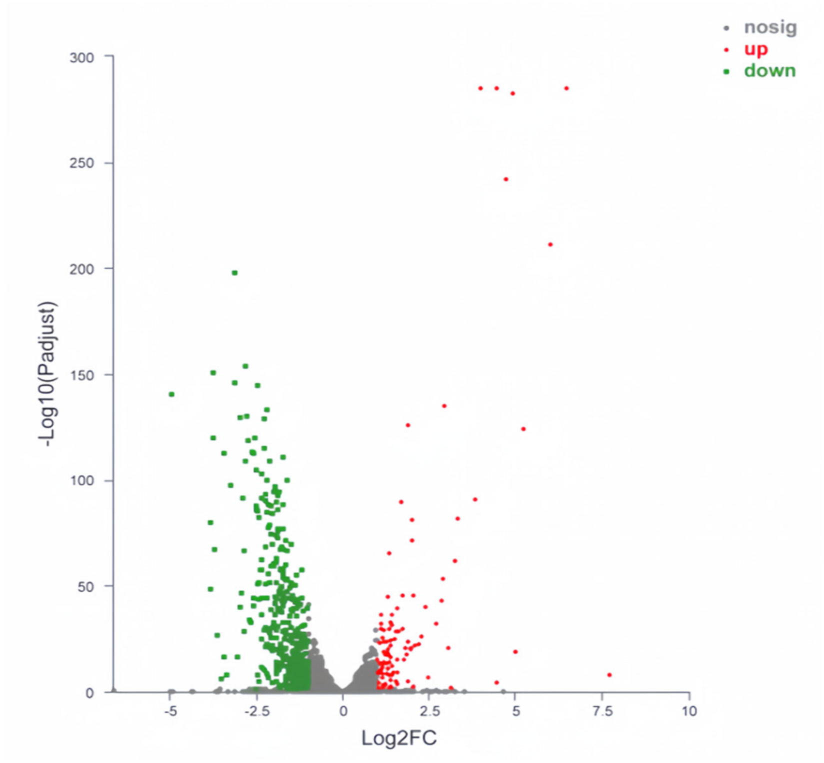

Figure S1. Volcano plot of differentially expressed genes under syringaldehyde stress.

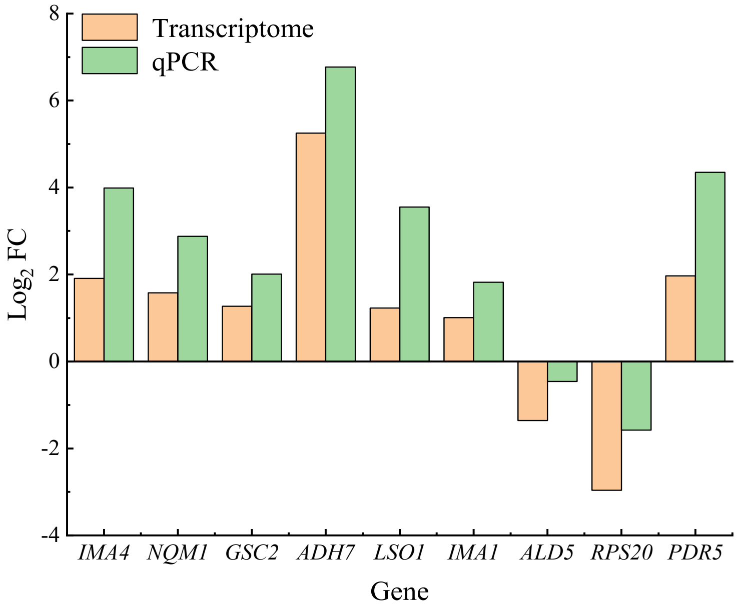

Figure S2. Comparison of RT-qPCR and RNA-seq expression patterns of selected differentially expressed genes.

Table S1. RT-qPCR Primer Sequences

Primer Name	Primer Sequence (5'→3')	Primer Name	Primer Sequence (5'→3')
IMA4-F	ACCTACGGTACGAACGAGGACTG	*IMA1-F*	GATGGCTGGGGTGACATGAAAGG
IMA4-R	TCGCTGGAGCAATGGTTGATGAC	*IMA1-R*	CTTGTGGCGAGTCGTAGAATGGTG
NQM1-F	GATAAGAAGGCGACGGTGAAGAAGG	*ALD5-F*	GGCTACAGGTAACACCGTCGTATTG
NQM1-R	ACCCTCCCACGTAGAAGCTATCTTG	*ALD5-R*	ATGCCTGCTTCCTGACACAACTG
GSC2-F	TGCTGACCCGTGAGTTCAAGAATG	*RPS20-F*	AGAATCACTTTGACCTCCACCAAGG
GSC2-R	CCTTGTCGGTTGAGTCCATGCC	*RPS20-R*	GACCAAGTTGTGTTGTTCAGCGTTC
ADH7-F	GCATCGGTATTTCCAACGCAAAGG	*PDR5-F*	GACTGTCTACTTTGGCGACTTGGG
ADH7-R	GATCCGCAGATACCACAGGCTTC	*PDR5-R*	TGGCGTCAGCAGGGCATTTATG
LSO1-F	AGAAGCTCAGAGGTGGGAAGAAGG	*ACT1-F*	GCCGAAAGAATGCAAAAGGA
LSO1-R	CTTCGGCGGCTAGTAATTGGTCTC	*ACT1-R*	TAGAACCACCAATCCAGACG
